# Modeling and Compensation of Dynamic Hysteresis with Force-Voltage Coupling for Piezoelectric Actuators

**DOI:** 10.3390/mi12111366

**Published:** 2021-11-05

**Authors:** Wen Wang, Jiahui Wang, Ruijin Wang, Zhanfeng Chen, Fuming Han, Keqing Lu, Chuanyong Wang, Zhenlong Xu, Bingfeng Ju

**Affiliations:** 1 School of Mechanical Engineering, Hangzhou Dianzi University, Hangzhou 310018, China; wangwn@hdu.edu.cn (W.W.); wangjiahui@hdu.edu.cn (J.W.); wangrjcn@hdu.edu.cn (R.W.); hfm@hdu.edu.cn (F.H.); lkq@hdu.edu.cn (K.L.); cywang@hdu.edu.cn (C.W.); xzl@hdu.edu.cn (Z.X.); 2 State Key Laboratory of Fluid Power Transmission and Control, Zhejiang University, Hangzhou 310027, China; mbfju@zju.edu.cn

**Keywords:** piezoelectric actuator, dynamic hysteresis, coupling hysteresis model, hysteresis compensation, Prandtl–Ishlinskii model

## Abstract

Piezoelectric actuators are widely used in the field of micro- and nanopositioning due to their high frequency response, high stiffness, and high resolution. However, piezoelectric actuators have hysteresis nonlinearity, which severely affects their positioning accuracy. As the driving frequency increases, the performance of piezoelectric actuators further degrades. In addition, the impact of force on piezoelectric actuators cannot be ignored in practical applications. Dynamic hysteresis with force-voltage coupling makes the hysteresis phenomenon more complicated when force and driving voltage are both applied to the piezoelectric actuator. Existing hysteresis models are complicated, or inaccurate in describing dynamic hysteresis with force-voltage coupling. To solve this problem, a force-voltage-coupled Prandtl–Ishlinskii (FVPI) model is proposed in this paper. First, the influence of driving frequency and dynamic force on the output displacement of the piezoelectric actuators are analyzed. Then, the accuracy of the FVPI model is verified through experiments. Finally, a force integrated direct inverse (F-DI) compensator based on the FVPI model is designed. The experimental results from this study show that the F-DI compensator can effectively suppress dynamic hysteresis with force-voltage coupling of piezoelectric actuators. This model can improve the positioning accuracy of piezoelectric actuators, thereby improving the working accuracy of the micro- or nano-operating system.

## 1. Introduction

Piezoelectric actuators are widely used in the field of micro-/nanopositioning with the continuous development of manufacturing [1] for products such as atomic force microscopes [2,3], fast tool servos (FTSs) [4,5], and piezoelectric inkjet printers [6,7]. Piezoelectric actuators have the advantages of high frequency response, high stiffness, and high resolution [8,9]. However, the inherent hysteresis characteristics of piezoelectric actuators seriously affect the positioning accuracy of the system [10]. As the driving frequency increases, the hysteresis becomes more serious [11,12]. In addition, the positioning accuracy of the piezoelectric actuator is affected by force in some applications such as using FTSs to process complex surfaces. Piezoelectric actuators generally use flexible hinges as guiding mechanisms. In this case, piezoelectric actuators are mainly subjected to force along the direction of expansion and deformation (the forces mentioned below are dynamic forces along the direction of expansion and deformation). As a result, the hysteresis phenomenon is more difficult to describe when force and driving voltage are applied to the piezoelectric actuator at the same time. This phenomenon is also defined as dynamic coupling hysteresis [13]. Therefore, it is necessary to analyze the influence of the driving voltage and force on the piezoelectric actuator, and to compensate for the hysteresis.

In order to describe the hysteresis of piezoelectric actuators, many researchers have proposed hysteresis models, including the Jiles–Atherton model [14,15,16], the Bouc–Wen model [17,18,19], the Preisach model [20,21,22], and the Prandtl–Ishlinskii model [23,24,25]. However, most of these models can only describe static hysteresis. Therefore, many dynamic hysteresis models have been proposed to solve this problem. Ben Mrad et al. [26] introduced the input voltage rate in the weight function, and proposed a rate-dependent Preisach model. Similar to this method, Al Janaideh et al. [27] proposed the rate-dependent Prandtl–Ishlinskii model by introducing the rate of input voltage into the threshold. Wang et al. [28] proposed the delay-play (D-Play) operator and established the dynamic delay Prandtl–Ishlinskii (DDPI) model. The D-Play operator introduces two delay coefficients in the play operator. The DDPI model can accurately describe high-frequency dynamic hysteresis. Some researchers have also used intelligent models to describe dynamic hysteresis, such as neural networks [29], fuzzy systems [30], etc. The accuracy of the above-mentioned dynamic models has been improved compared to the static hysteresis model.

However, these single-input dynamic hysteresis models are unable to describe the complex dynamic hysteresis with force-voltage coupling. Therefore, some researchers have carried out research on this issue. Dong et al. [31] proposed a dual-input Preisach model to describe the coupling effect of external load and driving voltage on the piezoelectric ceramic. Feng et al. [32] proposed a quality-related Prandtl–Ishlinskii model. This model can describe the hysteresis of the piezoelectric actuator caused by the load. However, it does not compensate for hysteresis. Zhou et al. [33] established a rate-dependent Prandtl–Ishlinskii model, and used a neural network to describe the cross-coupling effect on piezoelectric actuators due to the applied voltage and external load. Nevertheless, these models are more complicated or inaccurate in describing dynamic hysteresis with force-voltage coupling. Therefore, we aimed to research the influence of force and driving frequency on piezoelectric actuators, and to compensate for dynamic hysteresis with force-voltage coupling.

In this paper, first, an experimental platform is designed to research dynamic hysteresis with force-voltage coupling. Then, a force voltage coupled Prandtl–Ishlinskii (FVPI) model is proposed. This model characterizes the force-voltage coupling hysteresis of piezoelectric actuators. Finally, the inverse model is derived based on the FVPI model. A force integrated direct inverse (F-DI) compensator is designed and a direct inverse (DI) compensator based on the DDPI model is used for comparison experiments. The results from this study show that the inverse compensator effectively suppresses dynamic hysteresis with force-voltage coupling. Additionally, compared with the existing models, the FVPI model is more accurate than the DDPI model.

The remaining content of this paper is structured as follows: Section 2 introduces the composition of the experimental setup and discusses the effects of frequency and force on piezoelectric actuators; Section 3 proposes the FVPI model based on the DDPI model, and verifies the accuracy of the model through experiments. The comparison verifies the effectiveness of the force integrated direct inverse compensator in Section 4. Section 5 discusses the advantages of the FVPI model and experimental results of the compensation experiment; Section 6 comprises the conclusion.

## 2. Experimental Setup and Hysteresis Characteristics

### 2.1. Experimental Setup

**Figure 1:**
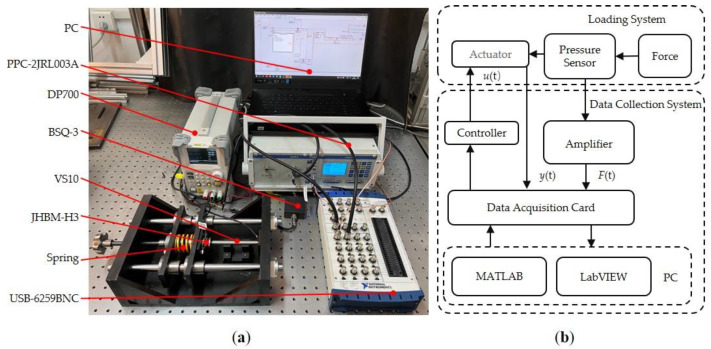
Experimenting Setup: (**a**) experimental equipment; (**b**) data acquisition system.

Figure 1a shows the experimental setup. The piezoelectric actuator (PSt150/5/100 VS10, Core Tomorrow, Harbin, China) has a working range of 0–95 µm, a stiffness of 5 N/µm, and a nominal thrust of 550 N. The piezoelectric actuator integrates a resistance strain gauge displacement sensor. The voltage output of the sensor module is 0–10 V. The output voltage resolution of the precision positioning controller (PPC-2CR0150, Maikerong, Suzhou, China) is 5 mV and the sensor module sensitivity is 3 µm/V. The range of the pressure sensor (JHBM-H3, Zhongwan Jingnuo, Bengbu, China) is 0–50 N, and the output sensitivity is 1.3 mV/V. The output voltage range of the signal amplifier (BSQ-3, Zhongwan Jingnuo, Bengbu, China) is 0–5 V. The maximum sampling rate of the data acquisition card (USB-6259BNC, National Instruments, Austin, TX, USA) is 1.25 Ms/s. The output voltage of the DC power supply (DP7000, RIGOL, Beijing, China) is 10V. Table 1 shows the performance parameters of the compression spring used. The software part of the data acquisition system was built by LabVIEW of PC.

Figure 1b shows the data acquisition system. The reaction force generated by the deformation of the compression spring is applied to the piezoelectric actuator. The compression spring deformation increases when the piezoelectric actuator squeezes the compression spring forward. As a result, the force gradually increases. On the contrary, the backward movement of the piezoelectric actuator results in a reduction in the compression spring deformation, resulting in a gradual decrease in the force.

**Figure 2:**
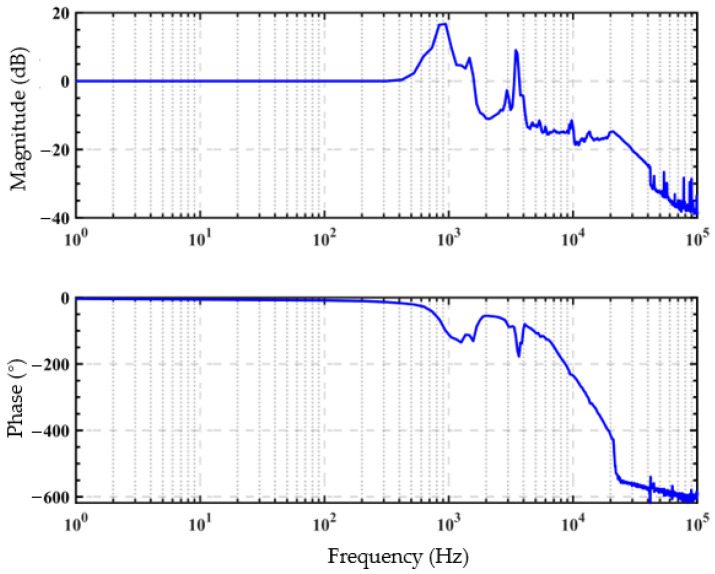
Experimental platform performance test.

The piezoelectric actuator is subjected to different dynamic forces by replacing the system with compression springs of different stiffnesses. The dynamic force can be measured by the pressure sensor. The signal of the pressure sensor is amplified by the signal amplifier. The displacement signal of the piezoelectric actuator and the pressure signal are collected by a data acquisition card. Finally, these two signals are transmitted to the computer for data processing and analysis to research dynamic hysteresis with force-voltage coupling. To obtain the bandwidth of the open-loop system, a sweep frequency experiment was conducted in this study. As shown in Figure 2, the resonant frequency of the open loop system is about 900 Hz. The magnitude is less affected at a frequency of 1–300 Hz; therefore, we chose to experiment within the frequency range of 1–300 Hz.

### 2.2. Hysteresis Characteristics

#### 2.2.1. Frequency Influence

**Figure 3:**
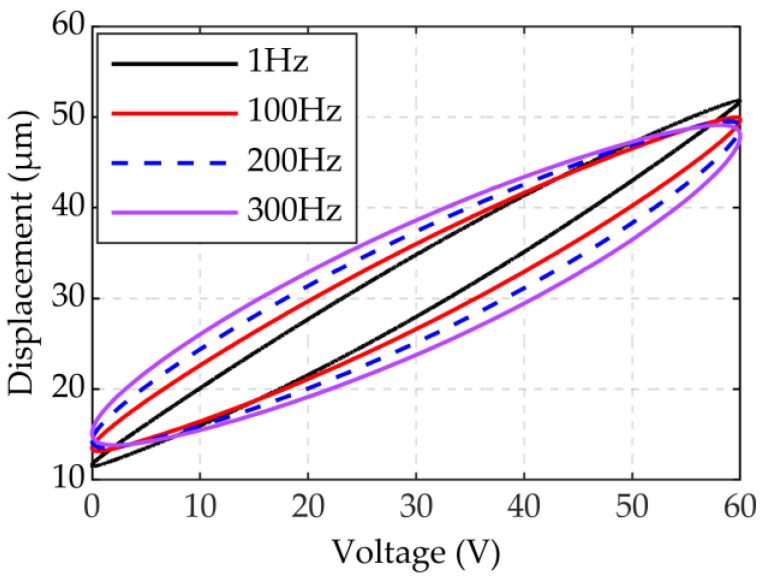
Influence of different frequencies on output displacement.

To research the influence of driving frequency on output displacement, the experiment was set up with the piezoelectric actuator placed horizontally on the support base without force. The driving voltage was set to *u*(*t*) = 30sin(2*πft* − *π*/2) + 30 (*f* = 1, 100, 200, and 300 Hz). The experimental results shown in Figure 3 indicate that the width of the hysteresis loop gradually increases as the driving frequency increases. However, the amplitude of the displacement is reduced. This phenomenon is called dynamic hysteresis and it is characteristic of piezoelectric actuators [34].

#### 2.2.2. Force Influence

Three groups of experiments were carried out on the experimental platform to research the influence of dynamic forces on piezoelectric actuators (case1: *u*(*t*) = 30sin(2*πft* − *π*/2) + 30, *f* = 1 Hz; case2: *u*(*t*) = 30sin(2*πft* − *π*/2) + 30, *f* = 25 Hz; case3: *u*(*t*) = 30sin(2*πft* − *π*/2) + 30, *f* = 250 Hz). These experiments were performed with different compression springs (spring 1, spring 2, spring 3, or spring 4). The preload applied to the piezoelectric actuator was 120 ± 0.5 N, which was adjusted by the handwheel and measured by the pressure sensor.

**Figure 4:**
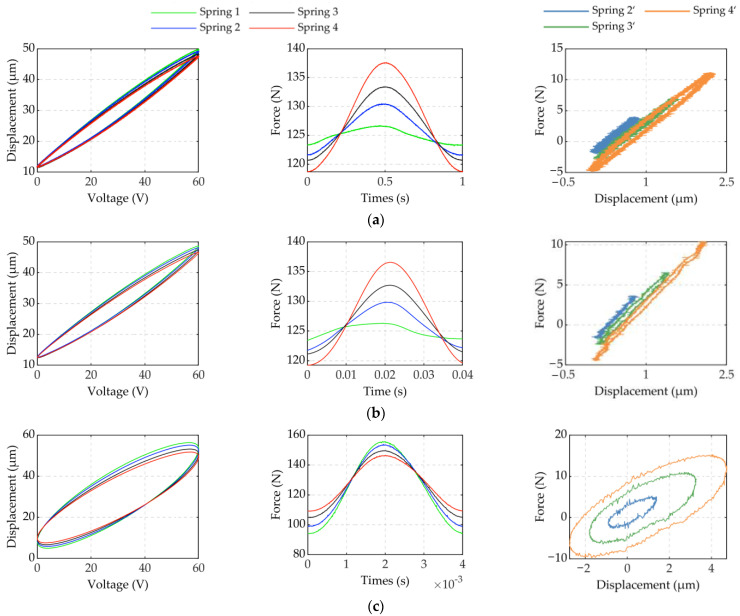
The influence of different dynamic forces: (**a**) 1, (**b**) 25, and (**c**) 250 Hz.

The voltage and displacement curves in Figure 4 show that the width of the hysteresis loop decreases with an increase in the dynamic force. Hysteresis becomes more severe as frequency and dynamic force increase. The hysteresis loop width changes as shown in Table 2. This is the coupling effect of frequency and dynamic force.

The pressure graph in Figure 4 shows that the increase in frequency influences the inertial force to have a greater impact on the performance of the pressure sensor. To obtain an accurate dynamic force, the force under the experimental conditions of Spring 2, Spring 3, and Spring 4 was subtracted from the dynamic force under the experimental conditions of spring 1. Compared with other compression springs, the stiffness of the Spring 1 is extremely small, which can be regarded as a situation without force. The exact force obtained is represented as Spring 2′, Spring 3′, and Spring 4′ in Figure 4. The relationship between dynamic force and changing displacement is shown in Figure 4 (the changing displacement refers to the difference between the displacement under the current spring and the displacement under spring 1). There is obvious hysteresis nonlinearity between dynamic force and changing displacement. Moreover, the hysteresis is more complicated and difficult to describe as the drive frequency increases. This is similar to the dynamic hysteresis of piezoelectric actuators. Therefore, it was necessary to establish a model that can accurately describe dynamic hysteresis with force-voltage coupling.

## 3. Hysteresis Model

### 3.1. Dynamic Delay Prandtl–Ishlinskii (DDPI) Model

The DDPI model [28] is composed of multiple D-Play operators weighted and superimposed. This model is often used to describe dynamic hysteresis. The discrete expression of the DDPI model is as follows:

(1) $$\{\begin{array}{l} F_{r,\tau ,\varphi }[u](k)=\max(u(k-\varphi )-r,\min(u(k-\tau )+r,F_{r,\tau ,\varphi }[u](k-1)))\\ y(k)=p_{0}u(k)+\sum_{i=1}^{n}p_{r}(r_{i})F_{r_{i},\tau ,\varphi }[u](k) \end{array}$$

where *y*(*k*) is the output displacement of the model at moment *k*, *F_r,τ,φ_*[*u*](*k*) is the output of the D-Play operator at moment *k*, *F_r,τ,φ_*[*u*](*k* − 1) is the output of the D-Play operator at moment *k* − 1, *u*(*k*) is the input voltage at moment *k*, *r_i_* is the threshold of the operator, *n* is the number of thresholds, *p*_0_ is the linear coefficient, and *p_r_* is the weight coefficient.

### 3.2. Force Voltage Coupling Prandtl–Ishlinskii (FVPI) Model

#### 3.2.1. Modified Model

Currently, the single-input DDPI model cannot describe dynamic hysteresis with force-voltage coupling. Therefore, a dual-input FVPI model needs to be proposed to improve the accuracy of the model.

The FVPI model is as follows:

(2) $$\{\begin{array}{l} y[u,F](k)=y_{1}[u](k)-y_{2}[F](k)\\ f_{r}[u](k)=\max\left\{u(k-\tau_{1})-r,\min\left\{u(k-\varphi_{1})+r,f_{r}[u](k-1)\right\}\right\}\\ y_{1}[u](k)=p_{0}u(k)+\sum_{i=1}^{n}w_{i}(r_{i})f_{r_{i}}[u](k)\\ f_{r}[F](k)=\max\left\{F(k-\tau_{2})-r,\min\left\{F(k-\varphi_{2})+r,f_{r}[F](k-1)\right\}\right\}\\ y_{2}[F](k)=p_{1}F(k)+\sum_{i=1}^{n}w_{Fi}(r_{i})f_{r_{i}}[F](k) \end{array}$$

where *y*(*k*) is the output displacement of the piezoelectric actuator at moment *k*; *u*(*k*) is the input voltage at moment *k*; *F*(*k*) is force at moment *k*; *y*_1_[*u*](*k*) is the output displacement only affected by the driving voltage at moment *k*; *y*_2_[*F*](*k*) is the output displacement affected by force at moment *k*; *f_r_*[*u*](*k*) is the output of the operator at moment *k*; *f_r_*[*F*](*k*) is the output of the operator at moment *k*; *f_r_*[*u*](*k* − 1) is the output of the operator at moment *k* − 1; *f_r_*[*F*](*k* − 1) is the output of the operator at moment *k*−1; *p*_0_ and *p*_1_ are linear coefficients; *w_i_* and *w_F_* are the weight coefficients; and *τ*_1_, *τ*_2,_ *φ*_1,_ and *φ*_2_ are the delay coefficients, and are positive integers.

The weight coefficients *w_i_* and *w_F_*; delay coefficients *τ*_1_, *τ*_2,_ *φ*_1,_ and *φ*_2_; and linear coefficients *p*_0_ and *p*_1_ are identified by the differential evolution (DE) algorithm. The objective function is as follows [35]:

(3) $$E_{\mathrm{ratio}}=\frac{\sum_{k=1}^{n}\left|y(k)-Y(k)\right|}{\sum_{k=1}^{n}\left|y(k)-\bar{y}\right|}$$

where *y*(*k*) is the actual displacement at moment *k*, *Y*(*k*) is the output displacement of the FVPI model at moment *k*, and $\bar{y}$ is the average value of the actual displacement.

To verify the accuracy of the FVPI model, two groups of experiments were carried out including two single frequency sinusoidal signals.

In experiment 1, the input voltage was set to *u*(*t*) = 30sin(2*πft* − *π*/2) + 30 (*f* = 1 Hz, 100 Hz, 200 Hz, 300 Hz). In experiment 1, the piezoelectric actuator was not affected by force. The threshold was set to r = [0,6,12,18,24,30,36,42,48,56,60].

In experiment 2, the input voltage was set to: *u*(*t*) = 30sin(2*πft −* *π*/2) + 30 (*f* = 250 Hz, *t* = [0,0.004]). The threshold was set to r = [0,6,12,18,24,30,36,42,48,56,60]. Table 3 shows the results of the parameters identified by the DE algorithm in experiment 2. Experiment 2 was carried out under compression springs of different stiffness to verify the performance of the FVPI model in describing dynamic hysteresis with force-voltage coupling and the results were compared with the DDPI model.

#### 3.2.2. Experiment Results

**Figure 5:**
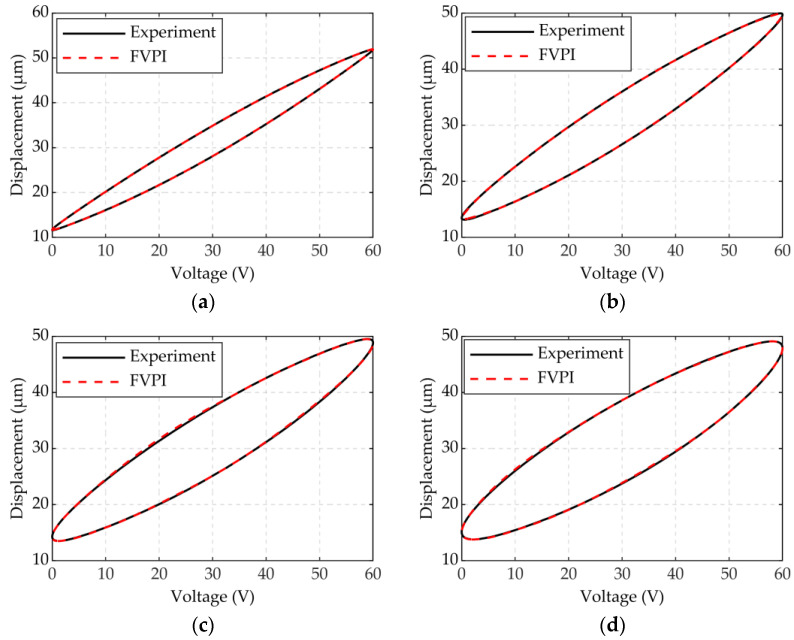
FVPI model with experiment at frequencies: (**a**) 1; (**b**) 100; (**c**) 200; (**d**) 300 Hz.

**Figure 6:**
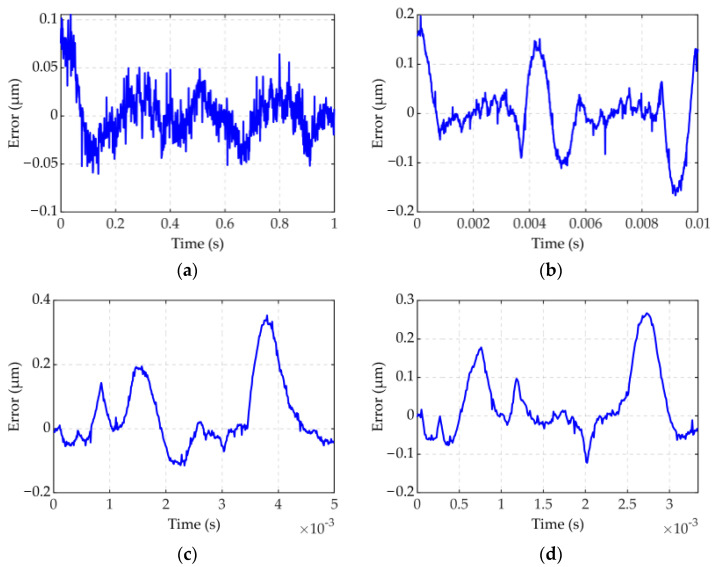
Model error at different frequencies: (**a**) 1; (**b**) 100; (**c**) 200; (**d**) 300 Hz.

**Figure 7:**
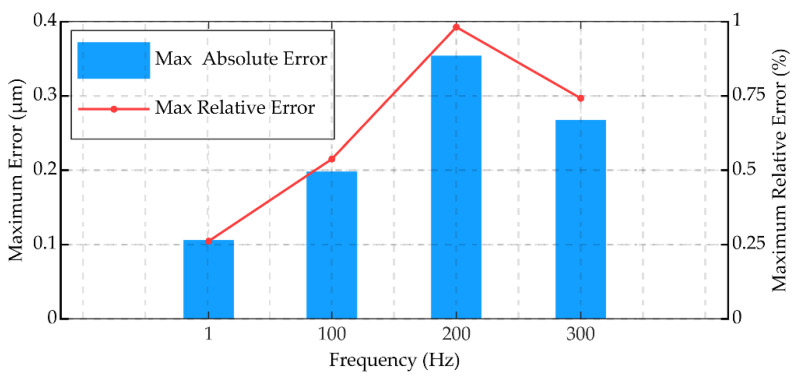
FVPI model error evaluation at different frequencies.

The result of experiment 1 is shown in Figure 5 and Figure 6. In Figure 5, the FVPI model describes the dynamic hysteresis of the piezoelectric actuator in 1–300 Hz without force. Figure 6 shows the error of the FVPI model. The results show that the FVPI model can accurately describe dynamic hysteresis. The maximum absolute error and maximum relative error were used to evaluate the accuracy of the FVPI model. Figure 7 shows that the maximum absolute error increased from 0.10 to 0.38 µm and the maximum relative error increased from 0.26% to 0.88% when the driving frequency increased from 1 to 300 Hz. The accuracy of the FVPI model decreased slightly with increasing frequency and the maximum relative error was below 1% in experiment 1.

**Figure 8:**
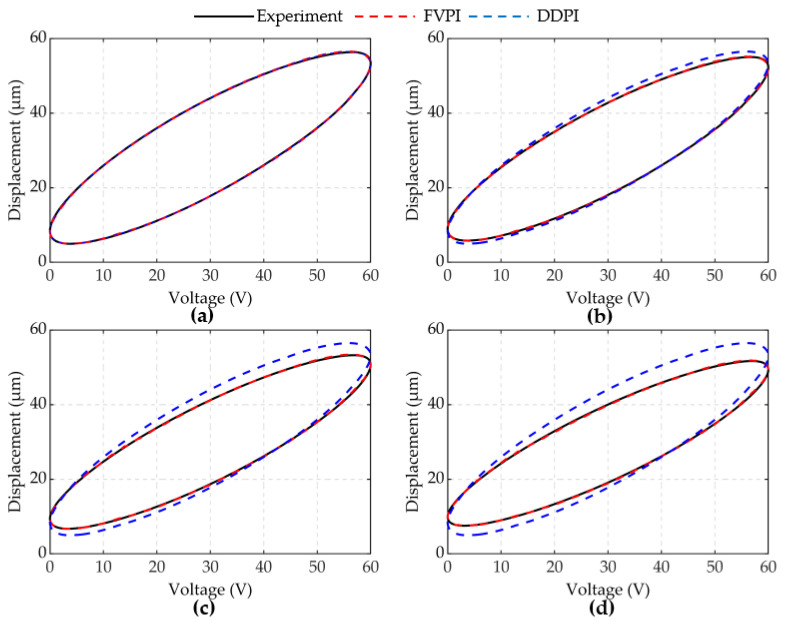
Comparison of two models at 250 Hz under different forces: (**a**) spring 1; (**b**) spring 2; (**c**) spring 3; (**d**) spring 4.

**Figure 9:**
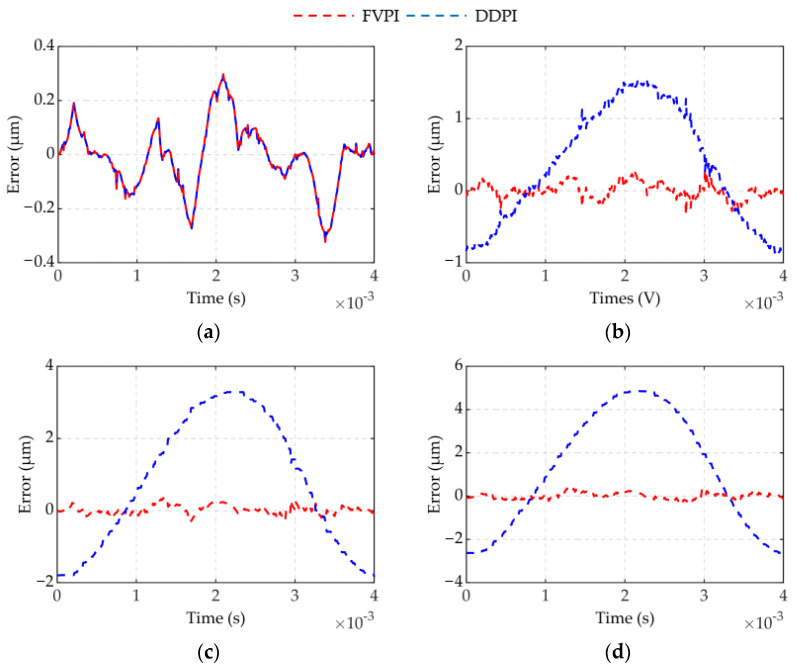
Error of two models at 250Hz under different loads: (**a**) spring 1; (**b**) spring 2; (**c**) spring 3; (**d**) spring 4.

**Figure 10:**
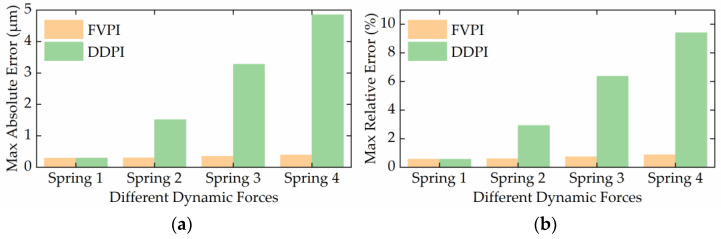
Two model error evaluation at different dynamic forces: (**a**) MAE; (**b**) MRE.

The results of experiment 2 are shown in Figure 8 and Figure 9. Figure 8 shows a comparison of the FVPI and DDPI models with the experimental data at 250 Hz driving frequency, under different forces. Figure 9 shows the error of the two models. The results show that the FVPI model can more effectively reduce the errors caused by force than DDPI model. Figure 10 shows that the maximum absolute error of the FVPI model increased from 0.30 to 0.39 µm as force increased. However, the output displacement amplitude of the piezoelectric actuator decreased when the force increased, resulting in a faster increase in the maximum relative error from 0.57% to 0.89%.

## 4. Feedforward Control

### 4.1. Feedforward Control Based on Inverse Model

**Figure 11:**
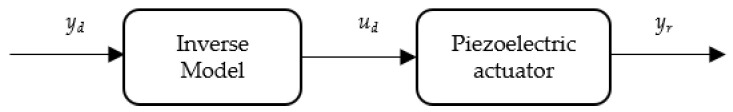
Schematic diagram of feedforward control.

Feedforward control based on the inverse model is an effective method to suppress the hysteresis nonlinearity of piezoelectric actuators [36]. As shown in Figure 11, to obtain the desired voltage *u_d_*, the desired voltage *y_d_* was put into the inverse model. Then, the expected voltage *u_d_* was applied to the piezoelectric actuator to realize linearization control. Therefore, it was necessary to derive the inverse model of the FVPI model to compensate for dynamic hysteresis with force-voltage coupling.

The play operator is related to the stop operator, as follows:

(4) $$f_{r}[u](k)+S_{r}[u](k)=u(k)$$

where *f_r_*[*u*](*k*) is the play operator, *S_r_*[*u*](*k*) is the stop operator, and *u*(*k*) is the input voltage at the moment *k*.

The stop operator of the FVPI model is as follows:

(5) $$S_{r,\tau }[y](k)=\min\left\{y(k)-y(k-\tau )+r,\max\left\{\begin{array}{l} y(k)-y(k-\varphi )-r,\\ y(k)-y(k-1)+S_{r,\tau }[y](k-1) \end{array}\right\}\right\}$$

where *S_r_*_,*τ*_[*y*](*k*) is the output of the stop operator at moment *k*, *S_r_*_,*τ*_[*y*](*k* − 1) is the output of the stop operator at moment *k* − 1, *y*(*k*) is the input displacement at moment *k*, *y*(*k* − 1) is the input displacement at moment *k* − 1, and *τ* and *φ* are the delay coefficients.

The inverse force voltage coupled Prandtl−Ishlinskii (IFVPI) model is as follows:

(6) $$\left\{ \begin{array}{l} y_{d2}(k)=p_{1}^{\prime }F(k)+\sum_{i=1}^{n}{w_{iF}}^{\prime }(r_{i})S_{r,\tau_{2},\varphi_{2}}[F](k)\\ y_{d}(k)=y_{d1}(k)+y_{d2}(k)\\ u_{d}(k)=p_{0}^{\prime }y_{d}(k)+\sum_{i=1}^{n}{w_{i}}^{\prime }(r_{i})S_{r,\tau_{1},\varphi_{1}}[y_{d}](k) \end{array}\right.$$

where *u_d_*(*k*) is the expected voltage at moment *k*; *y_d_*(*k*) is the exact expected displacement at moment *k*; *y_d_*_1_(*k*) is the expected displacement without force at moment *k*; *y_d_*_2_ (*k*) is the displacement affected by force at moment *k*; *p*_0_′, *p*_1_′ are linear coefficients; *w_i_*’(*r_i_*) and *w_iF_*’(*r_i_*) are the weight functions of the stop operator. *w_i_*’(*r_i_*), *w_iF_*’(*r_i_*), *p*_0_’, *p*_1_’, *τ*_1_, *τ*_2_, *φ*_1_, and *φ*_2_ were the parameters to be identified.

**Figure 12:**
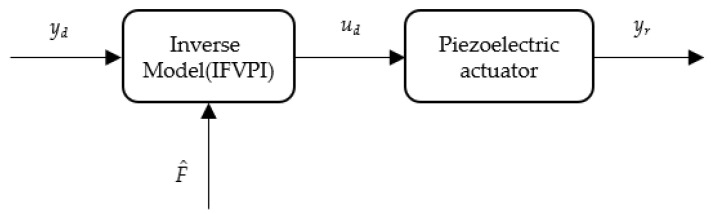
Schematic diagram of IFVPI feedforward control.

The feedforward control flow chart was modified as shown in Figure 12. Compared to the feedforward control of the single-input model, force is also input to the inverse compensation as a variable. The inverse compensator is defined as the force integrated direct inverse (F-DI) compensator. Feedforward control often needs to predict the input signal. Therefore, we used the estimated force $\hat{F}$ instead of the actual force *F*. The estimated force is as follows:

(7) $$\hat{F}=k_{i}y_{d}$$

where $\hat{F}$ is the estimated force, *k_i_* is the stiffness of the compression spring, and *y_d_* is the expected displacement.

Comparison experiments were carried out to verify the effectiveness of the force integrated direct inverse (F-DI) compensator. The direct inverse (DI) compensator used in the experiment is based on the DDPI model.

In experiment 1, the desired trajectory was set to *y_d_* = 20sin(2*πft* − *π*/2) + 30 (*f* = 100 Hz, *t* = [0,0.03]). The piezoelectric actuators were tested under the conditions of spring 2, spring 3, and spring 4.

In experiment 2, the desired trajectory was set to *y_d_* = 20sin(2*πft* − *π*/2) + 30 (*f* = 250 Hz, *t* = [0,0.012]). The piezoelectric actuators were tested under the conditions of spring 2, spring 3, and spring 4.

### 4.2. Results

**Figure 13:**
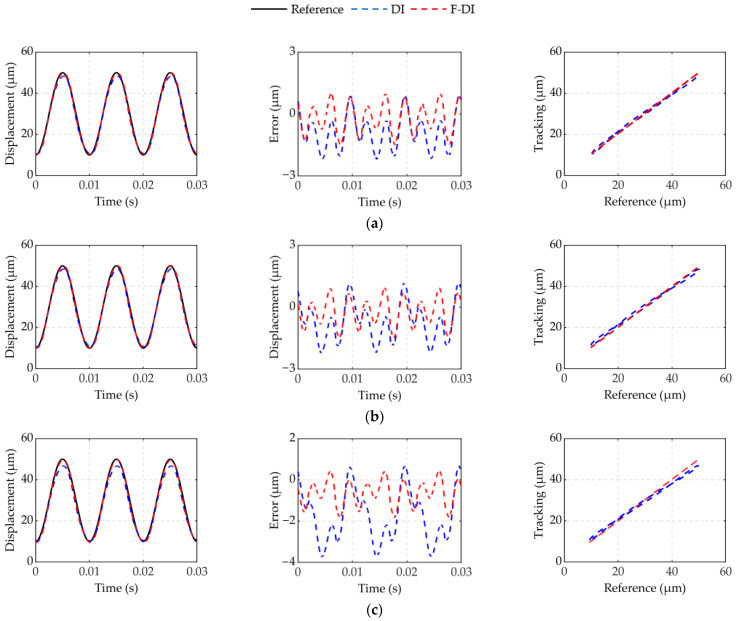
Feedforward experimental in 100 Hz at different forces: (**a**) spring 2; (**b**) spring 3; (**c**) spring 4.

**Figure 14:**
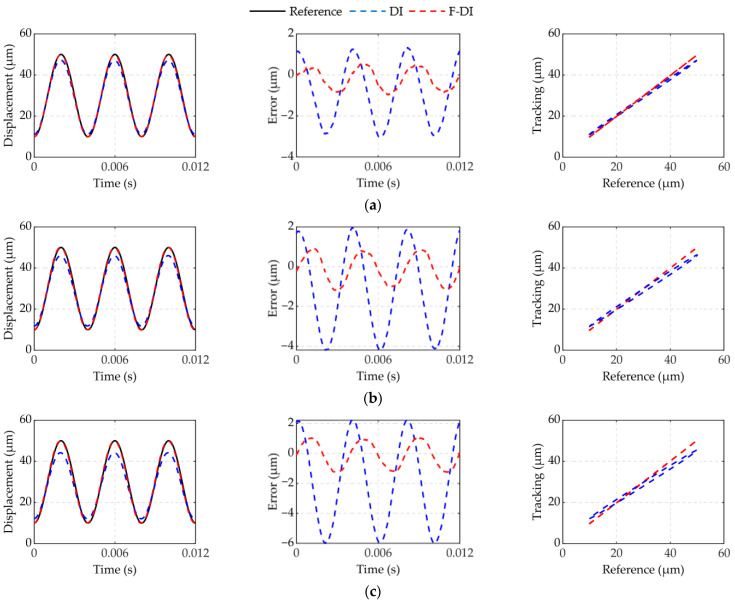
Feedforward experimental in 250 Hz at different forces: (**a**) spring 2; (**b**) spring 3; (**c**) spring 4.

Figure 13 and Figure 14 show the experimental results of feedforward compensation driven in 100 and 250 Hz under different forces. The experimental results show that the F-DI compensator can more effectively compensate for dynamic hysteresis with force-voltage coupling than the DI compensator. Compensators were evaluated using the maximum absolute error. Table 4 shows the maximum absolute error results of the F-DI compensator and the DI compensator in 100 and 250 Hz under different dynamic forces. The error of the F-DI compensator was reduced by 60% compared to the DI compensator. The effect of the DI compensator worsened with the increase in force and frequency. However, the F-DI compensator still had a higher compensation accuracy. In experiment 1, the maximum error was reduced from 9.3% to 4.6%. In experiment 2, the maximum error was reduced from 15% to 3.1%.

## 5. Discussion

Recently, piezoelectric actuators have been widely used in ultra-precision positioning systems as high-precision driving devices. However, piezoelectric actuators cannot meet high-precision positioning requirements due to the hysteresis of dynamic coupling caused by driving frequency and dynamic force. Therefore, this paper proposes a force voltage coupling model to describe dynamic hysteresis with force-voltage coupling. The F-DI compensator was designed based on the FVPI model to suppress the hysteresis nonlinearity of piezoelectric actuators. The findings in this paper may be beneficial in improving the positioning accuracy of piezoelectric actuators.

**Figure 15:**
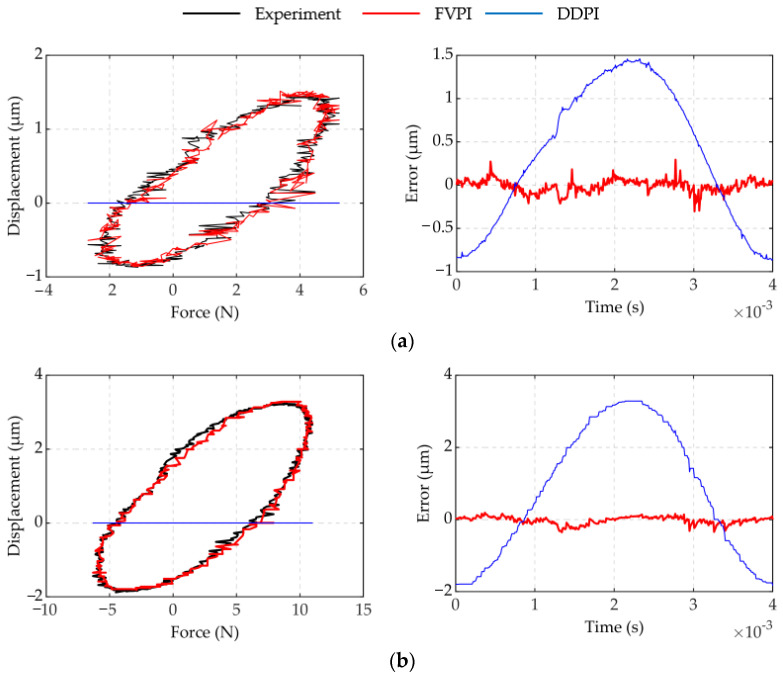

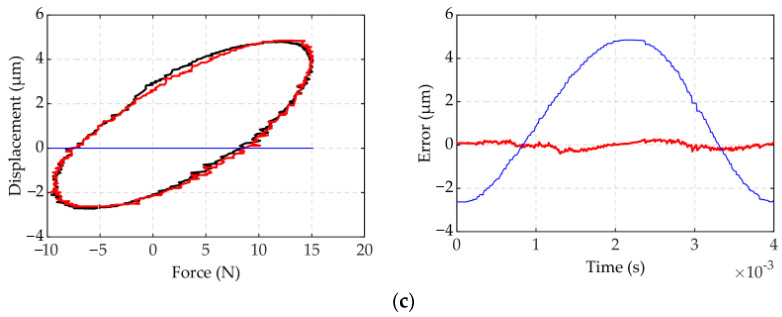
The FVPI model and experimental data at 250 Hz under different forces: (**a**) spring 2; (**b**) spring 3; (**c**) spring 4.

Most hysteresis models currently describe the hysteresis of piezoelectric actuators with no load. The accuracy of these models decreases faster when the force on the piezoelectric actuator increases. The DDPI model is a single-input model, which is not sensitive to the effects of force. As a result, it is not effective in describing and compensating for dynamic hysteresis with force-voltage coupling. Table 4 shows that as the frequency and force increase, the compensation effect worsens. The FVPI model is a dual-input model, which can describe dynamic hysteresis with force-voltage coupling by introducing two factors: force and voltage. The FVPI model not only inherits the superior performance of the DDPI model to describe high-frequency dynamic hysteresis, but also accurately describes the effect of force on the piezoelectric actuator. As shown in Figure 15, the FVPI model describes the hysteresis of piezoelectric actuators affected by dynamic force at 250 Hz. The experimental results show that the FVPI model has good accuracy even if the force signal fluctuates greatly. Signal fluctuations can cause difficulty in parameter identification and can reduce the accuracy of identification by introducing the rate of the input signal. However, signal fluctuations have little effect on parameter identification with the introduction of delay coefficients. In addition, the hysteresis caused by force is similar to dynamic hysteresis. These are the reasons why the FVPI model can accurately describe dynamic hysteresis with force-voltage coupling. Furthermore, in Figure 4, the phenomenon that the width of the hysteresis loop decreases with an increase in the dynamic force, is interesting. The dynamic force hinders the movement of the piezoelectric actuator and reduces its displacement amplitude, leading the rate of displacement to decrease. Normally, dynamic hysteresis is rate-dependent. It may be the reason for the phenomenon, which may help to compensate for the hysteresis of the piezoelectric actuator.

**Figure 16:**
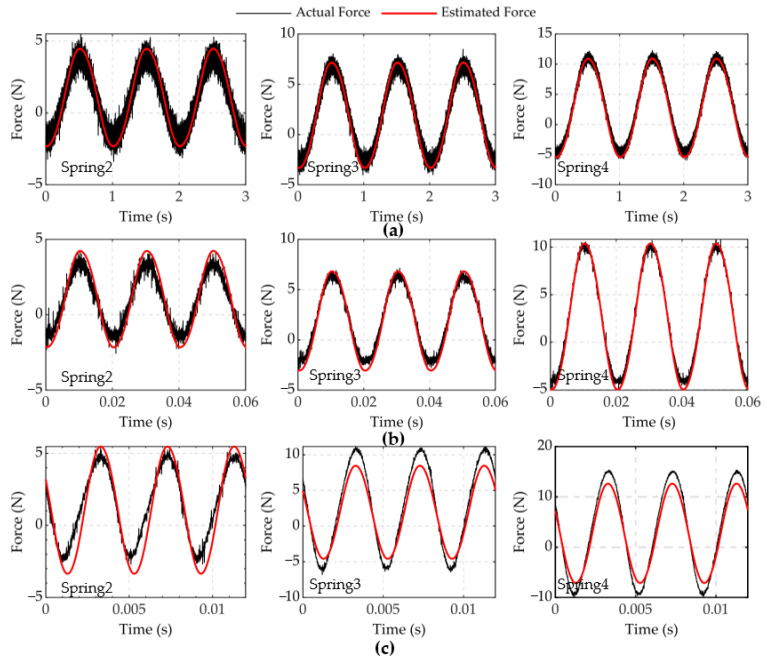
Comparison between the actual dynamic force and the estimated force at different frequencies: (**a**) 1; (**b**) 50; (**c**) 250 Hz.

However, the effect of the feedforward compensation experiment was not as accurate as expected. The accuracy of the input signal has an influence on the output displacement of the piezoelectric actuator, which reduces the accuracy of parameter identification. Figure 16 shows the comparison between the actual dynamic force and the estimated force at 250 Hz. The error between the estimated force and the actual force gradually increases with an increase in the driving frequency. The increased error leads to the inaccuracy of the FVPI model in forecasting, which affects the effect of feedforward compensation. Thus, for the follow-up research, it will be necessary to establish a more accurate force estimation model to improve the compensation accuracy of the feedforward experiment. More specifically, we will use feedback control to further improve the positioning accuracy of the piezoelectric actuator in the future.

## 6. Conclusions

Dynamic hysteresis with force-voltage coupling affects the positioning accuracy of piezoelectric actuators. In this paper, a FVPI model was proposed to accurately describe dynamic hysteresis with force-voltage coupling. Firstly, the influence of different driving frequencies and dynamic forces on the output displacement of the piezoelectric actuator was analyzed through experiments. Then, the FVPI model was established according to the law obtained from the experiment and was verified through experiments. Experimental results showed that the FVPI model can accurately describe dynamic hysteresis with force-voltage coupling of piezoelectric actuators. Moreover, the maximum relative error of the model accuracy was less than 1% in the 1–300 Hz range. Finally, the F-DI compensator was designed based on the FVPI model and compared with the DI compensator. The experimental results verified the effectiveness of the F-DI compensator and the positioning error of the F-DI compensator was reduced by 60% compared with the DI compensator.

## Figures and Tables

**Table 1:** Compression spring performance parameters.

Parameters	Spring 1	Spring 2	Spring 3	Spring 4
Outer diameter (mm)	40	40	40	40
Inner diameter (mm)	20	20	20	20
Length (mm)	40	40	40	40
Ultimate compression rate (%)	50	32	24	20
Ultimate pressure (N)	1254	3136	3364	4163
Stiffness (N/µm)	0.063	0.245	0.350	0.520

**Table 2:** Width of hysteresis loops under different dynamic forces.

Frequency	Spring 1	Spring 2	Spring 3	Spring 4
1 Hz	6.49 µm	6.33 µm	6.10 µm	5.85 µm
25 Hz	6.92 µm	6.74 µm	6.55 µm	6.23 µm
250 Hz	26.94 µm	25.83 µm	23.17 µm	21.59 µm

**Table 3:** The result of parameter identification.

*c*	*r_i_*	*ω_i_*	*ω_Fi_*	*p*_0_	*p*_1_	*τ*_1_	*τ*_2_	*φ*_1_	*φ*_2_
0	0	12.44	2.80 × 10^−3^	1.65	6.97 × 10^−3^	169	45	161	44
1	6	1.69	0.19						
2	12	0.23	0.03						
3	18	0.85	5.02 × 10^−14^						
4	24	0.53	5.44 × 10^−15^						
5	30	1.63	2.22 × 10^−15^						
6	36	0.67	1.03 × 10^−14^						
7	42	0.51	1.50 × 10^−14^						
8	48	0.57	1.88 × 10^−15^						
9	56	0.68	3.66 × 10^−15^						
10	60	1.75	0.56						

**Table 4:** Maximum error between F-DI compensator and DI compensator.

Variable	DI		F-DI	
Frequency	100 Hz	250 Hz	100 Hz	250 Hz
Spring 2	2.19 µm	3.02 µm	1.50 µm	0.96 µm
Spring 3	2.23 µm	4.19 µm	1.52 µm	1.18 µm
Spring 4	3.72 µm	6.00 µm	1.85 µm	1.25 µm
